# The Role of Epilepsy Surgery in the Treatment of Childhood Epileptic Encephalopathy

**DOI:** 10.1155/2013/983049

**Published:** 2013-04-18

**Authors:** Husam R. Kayyali, Ahmed Abdelmoity, Saleh Baeesa

**Affiliations:** ^1^Department of Neurosciences, King Faisal Specialist Hospital and Research Center, Jeddah 21499, Saudi Arabia; ^2^Department of Neurology, Children's Mercy Hospital and Clinics, Kansas City, MO 64108, USA; ^3^Division of Neurosurgery, College of Medicine, King Abdulaziz University, Jeddah 21589, Saudi Arabia; ^4^Division of Neurological Surgery, King Abdulaziz University Hospital, P.O. Box 80215, Jeddah 21589, Saudi Arabia

## Abstract

Children with epileptic encephalopathy often have global impairment of brain function and frequent intractable seizures, which contribute further to their developmental disability. Many of these children have identifiable brain lesion on neurological imaging. In such cases, epilepsy surgery may be considered as a treatment option despite the lack of localized epileptic pattern on electroencephalogram (EEG). In this paper, we summarize the clinical features of epileptic encephalopathy syndromes and review the reported literature on the surgical approach to some of these disorders.

## 1. Introduction

Epileptic encephalopathy is defined as a condition in which the epileptiform abnormalities themselves are believed to contribute to the progressive disturbance in cerebral function [1]. The report of the International League Against Epilepsy (ILAE) Task Force on Classification and Terminology includes eight syndromes under epileptic encephalopathies. One common feature among these epilepsy syndromes is the suboptimal response to treatment with antiepileptic medications. This invited the utilization of epilepsy surgery in selected patients who have structural brain lesion believed to be the cause of epilepsy. 

In this paper, we briefly review the clinical features of different epileptic encephalopathy syndromes and summarize the reported literature on the surgical approach and management of some of these disorders.

## 2. Classification of Epileptic Encephalopathies 

According to the age of onset, epileptic encephalopathy syndromes may be divided into two main groups.

### 2.1. Infantile Epileptic Encephalopathies

#### 2.1.1. Ohtahara Syndrome

 First described in 1976 by Ohtahara, Ohtahara Syndrome is characterized by tonic seizures and burst suppression pattern on EEG [2]. Symptoms develop earlier than other forms of epileptic encephalopathies within the first 3 months of life, usually in the first 10 days. Etiology is unclear but it generally accompanies structural brain anomalies. Seventy-five percent of cases turn into West syndrome within 3 to 6 months, and some of these turn into Lennox-Gastaut syndrome. Seizures are resistant to treatment and generally have a poor prognosis.

#### 2.1.2. Early Myoclonic Encephalopathy

Has an early onset within the first few months of life in the form of erratic, fragmentary, or massive myoclonic seizures. Frequency varies from occasional to almost continuous myoclonus. Infants have severe delay in development, hypotonia, and disturbed alertness, sometimes with vegetative state. EEG is characterized by a burst-suppression pattern. Erratic myoclonus does not generally have an ictal EEG counterpart. Etiology remains often unknown. Some inborn errors of metabolism were suggested such as nonketotic hyperglycinemia, propionic acidemia, molybdenum cofactor deficiency, and methylmalonic acidemia. Cerebral malformations can also cause early myoclonic encephalopathy, but more often they produce Ohtahara syndrome [3]. The prognosis is poor since there is no effective therapy.

#### 2.1.3. West Syndrome

It usually occurs in the first year of life and consists of the triad of infantile spasms, developmental deterioration, and hypsarrhythmia pattern on EEG [4]. There is a broad range of potential causes, including cerebral malformations, infection, hemorrhage, hypoxic ischemic injury, metabolic disorders, and genetic conditions, such as Down syndrome [5]. No clear etiology is found in approximately 25–40% of cases. Adrenocorticotropin hormone (ACTH) and vigabatrin are widely used for treatment with variable degrees of success depending on the etiology. The ketogenic diet was found to be helpful in some cases [6]. Focal cortical resection or hemispherectomy may be considered for cases that are lesional and medically intractable [7]. The developmental prognosis depends partially on the etiology; normal development was described in 51% of cryptogenic cases versus only 6% of symptomatic cases. 

#### 2.1.4. Severe Myoclonic Epilepsy in Infancy (Dravet Syndrome)

It presents typically with frequent myoclonic seizures in the first year of life. They are often associated with fever and involve one side of the body although both sides of the body may be involved [8]. During the second year of life seizures become more persistent and no longer occur in association with fever. The early development of affected children is usually normal, but during the second year of life developmental regression occurs affecting mainly language and cognitive skills. On EEG there are spike and wave or polyspike discharges, which may be generalized or regional. 35–40% of patients have mutation of the SCN1A gene [9]. Seizures are very resistant to antiepileptic drugs. A combination of sodium valproate with either topiramate or stiripentol may be the most helpful. A short course of prednisolone and the ketogenic diet may also be helpful. Children with Dravet syndrome continue to have severe developmental disabilities and learning difficulties requiring full educational support.

### 2.2. Childhood Epileptic Encephalopathies

#### 2.2.1. Lennox-Gastaut Syndrome (LGS)

It is characterized by multiple seizure types, mental retardation or regression, and characteristic findings on EEG with paroxysms of fast activity and generalized slow spike and wave discharges (1.5–2 Hz). Seizure onset is usually at 1–8 years, peaking between 3 and 4 years. The most common seizure types are tonic, atonic, and atypical absence seizures, but myoclonic and generalized tonic-clonic seizures can be observed [10, 11]. According to etiology it is divided into cryptogenic or symptomatic. Symptomatic cases may be secondary to hypoxic ischemic encephalopathy, congenital brain malformation, vascular malformation, genetic conditions like tuberous sclerosis, trauma, brain tumor, or perinatal meningoencephalitis [12]. Antiepileptic medications, ketogenic diet, and hormonal therapies are used in treatment with variable success. Surgical treatment has been suggested for patients with structural brain lesions as discussed below. 

#### 2.2.2. Electrical Status Epilepticus during Slow Sleep (ESES)

It is a disorder that includes clinical manifestations of variable seizure types, deterioration of neuropsychological functions, and typical EEG pattern of continuous spikes and waves during slow sleep [13]. The age of onset ranges between 2 months and 12 years, with a peak around 3 to 5 years. Etiology is often unclear. Brain MRI shows diffuse or unilateral atrophy in 33% of cases [14]. Seizures may become self-limited and disappear in the midteens. However, many of affected children do not return to normal levels, particularly in the verbal area and attention [15].

#### 2.2.3. Landau-Kleffner Syndrome (LKS)

It is also known as acquired epileptic aphasia since this is the main clinical feature of this syndrome in addition to the presence of frequent spikes in the temporal or centrotemporal region activated during sleep. Onset is between 2 and 7 years in children with previously normal development [16]. It is more common in males. The presence of normal development in premorbid period is an important feature; however, preexisting language anomaly was described in 13% of cases [17]. Many therapeutic modalities have been tried with variable success. Among these are anticonvulsants, corticosteroids, IV immunoglobulin, ketogenic diet, and surgical intervention with multiple subpial transactions (MSTs) [18].

## 3. Surgical Approach to Children with Epileptic Encephalopathy

Epilepsy surgery was originally introduced as a treatment modality for patients who had localized epileptiform discharges on EEG. These findings are important clues to the cortical region that has to be removed to stop the seizures, and they remain a cornerstone of selection for surgery in most cases. However, over the years and with the advances achieved in neuroimaging, workers in this field developed more comprehensive approach to these patients, and the plan for epilepsy surgery nowadays is built on data gathered from EEG, magnetic resonance imaging (MRI), positron emission tomography (PET), single-photon emission-computed tomography (SPECT), neurologic examination, and seizure semiology [19]. The level of concordance between these different sources of data correlates with postsurgical seizure freedom rates.

EEG in patients with epileptic encephalopathy due to congenital or early acquired brain lesion may sometimes reveal diffuse or bilaterally distributed multifocal epileptiform activity. “The exception to the rule” for surgical candidacy despite generalized or bilateral multifocal EEG features is based on the experience gained during infancy and early childhood, when the age-related pattern of hypsarrhythmia may manifest in response to a variety of diffuse or focal brain insults or lesions [7]. Furthermore, it has been noted that localized cortical abnormalities may occasionally cause generalized epilepsies such as Lennox-Gastaut syndrome [19]. The exact mechanisms behind these phenomena are unknown, but the generalized and contralateral epileptiform discharges may be a manifestation of potentially reversible secondary epileptogenesis resulting from an interaction between the early lesion and the developing brain [20] although further research is needed to refine this understanding.

Based on the above, it has been proposed that infants and young children with intractable epilepsy and focal brain lesion may be candidates for epilepsy surgery despite the presence of generalized EEG seizures and a diffuse pattern of multifocal or bilateral epileptiform discharges [7, 21, 22].

### 3.1. Case Illustrations

The following two examples illustrate the successful utilization of epilepsy surgery in such clinical scenarios.

**Case 1**

**Figure 1:**
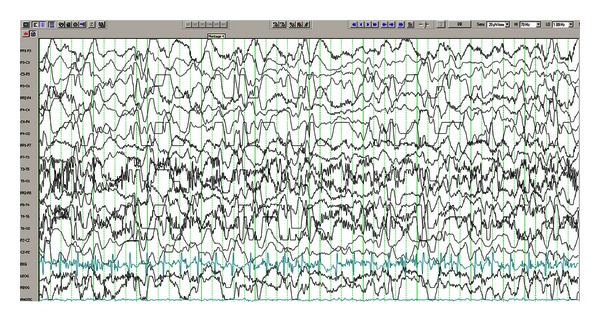
EEG prior to surgery showing generalized hypsarrhythmia.

**Figure 2:**
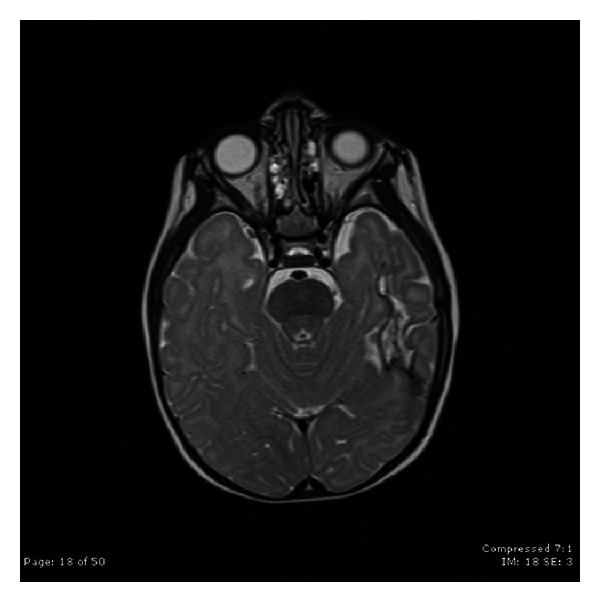
Axial T2-WI MRI scan demonstrating congenital vascular malformation in the left temporal lobe.

**Figure 3:**
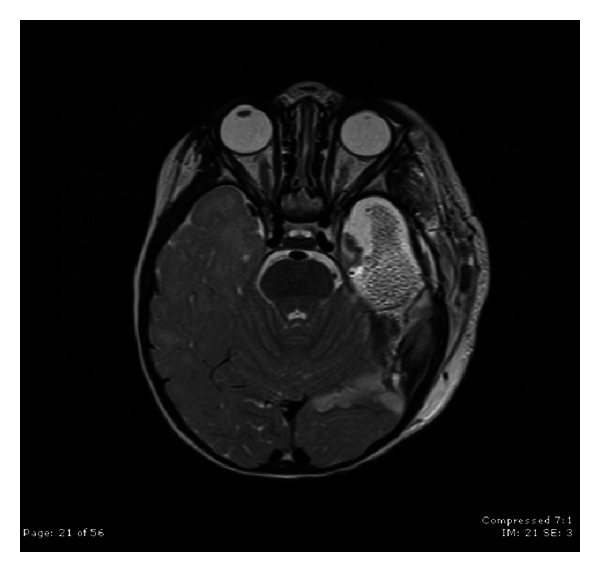
Postoperative axial T2-WI MRI scan at the same level after left temporal lobe resection.

A 7-month-old infant girl was born at full term after uncomplicated pregnancy and normal delivery and presented with increasing number of epileptic spasms at age 3 months. She had an initial normal development. EEG confirmed the presence of hypsarrhythmia pattern (Figure 1). Brain MRI showed vascular malformation in the left temporal lobe (Figure 2). Conventional antiepileptic treatment was initially tried using adrenocorticotropin hormone (ACTH) then topiramate. This resulted in partial control of her spasms. Then at age of 11 months it was determined that surgical resection of the left temporal vascular lesion would be beneficial since the child failed medication therapy and the vascular malformation carries a risk of intracranial hemorrhage (Figure 3). The child became seizure-free after surgery despite weaning off all antiepileptic medications 6 months later. Developmentally she made remarkable progress after the cessation of her seizures. Three years after surgery she remains seizure-free and developing normally for her age.

**Case 2**

**Figure 4:**
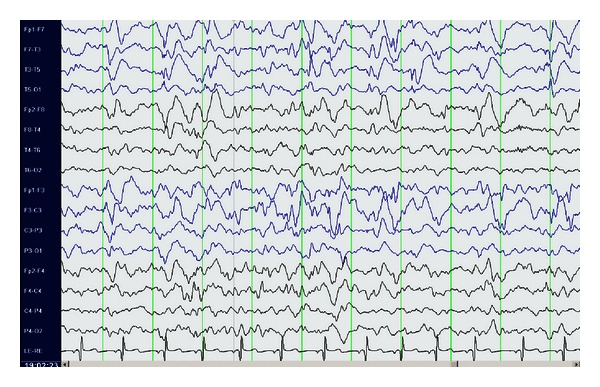
EEG prior to surgery showing multifocal sharp waves in the left and right temporal and frontal regions, maximal on the left side.

**Figure 5:**
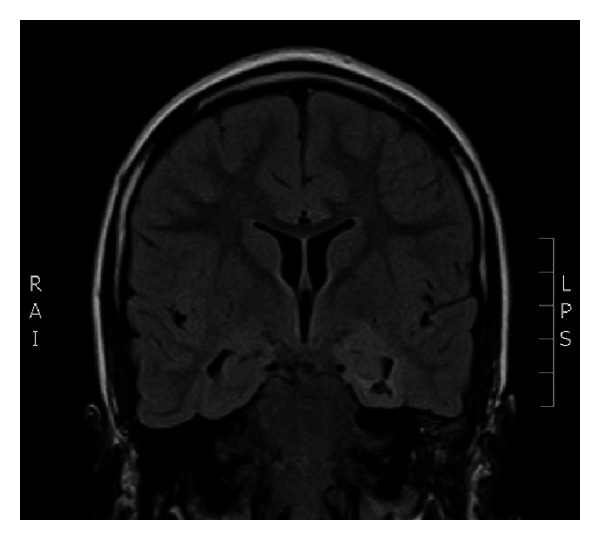
Coronal FLAIR MRI scan demonstrating the left mesial temporal lesion.

**Figure 6:**
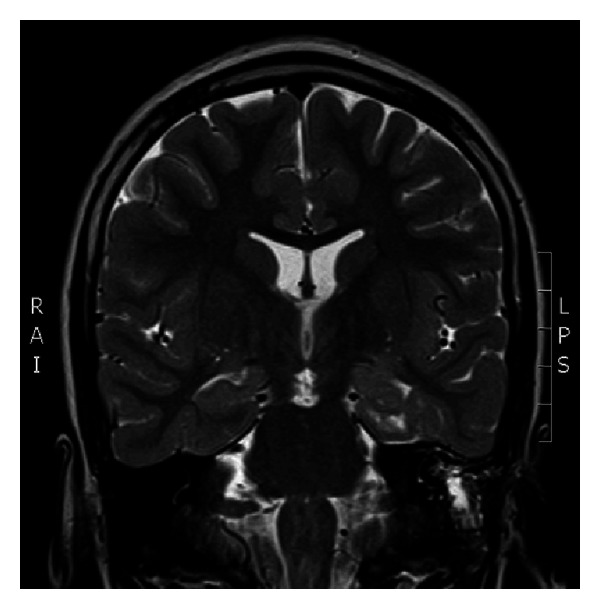
Coronal T2-WI MRI scan demonstrating the left mesial temporal lesion.

A 9-year-old ambidextrous girl presented with intractable epilepsy since age 2 years. Since the onset of her seizures she had global delay of her development affecting mainly communication, social, and cognitive skills. Despite treatment with large number of antiepileptic medications she continued to have 5–10 seizures daily. Her seizures start with an aura (sensation of fear) followed by tonic posturing of the upper body with eyes and head deviation to the right side, and then she has clonic jerking of the right arm and leg. Some of her seizures involve left hand dystonic movement and secondary generalization. Interictal EEG showed multifocal sharp waves in the left and right temporal and frontal regions. However, majority of sharp waves were in the left temporal region (Figure 4). Ictal onset was in the left frontotemporal region in three of the recorded seizures. The other two seizures were difficult to lateralize on scalp EEG. Brain MRI showed nonenhancing lesion in the left mesial temporal region, which was hyperintense on T2 images (Figures 5 and 6).

**Figure 7:**
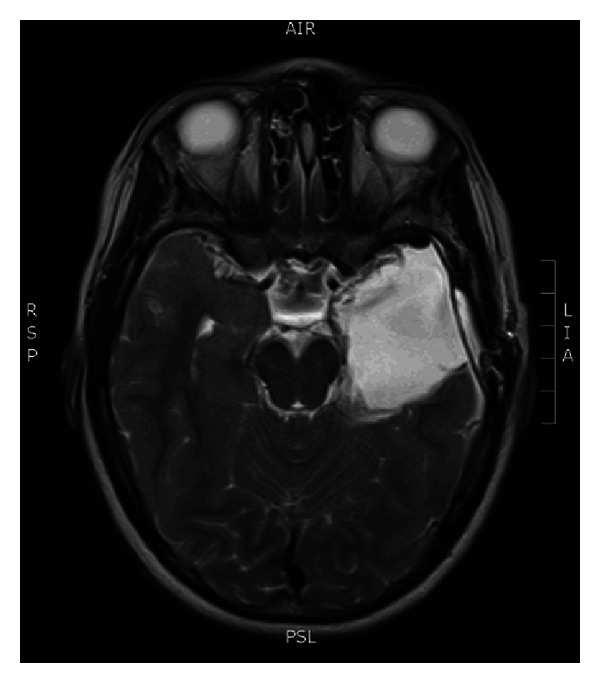
Postoperative axial T2-WI MRI scan at the same level after left temporal lobe resection.

Even though scalp EEG provided an evidence of multifocal bilateral epileptic process, the decision was made to proceed with left mesial temporal resection based on the following: (1) the presence of left mesial temporal lesion, (2) majority of epileptic activity was recorded from the left temporal region, (3) seizures were resistant to treatment with antiepileptic medications, and (4) patient's severe epilepsy caused significant global cerebral dysfunction, and she was not expected to have further deficit as a result of the planned surgery. The resection was done without any major complications (Figure 7). The pathology showed ganglioglioma (WHO grade I). On her four-month followup after surgery, the patient remained seizure-free, and she had remarkable improvement of her level of function. 

## 4. Surgical Outcome of Children with Epileptic Encephalopathy in the Literature

In 2007, Wyllie et al. reported 50 pediatric patients with intractable epilepsy since early in life, developmental delay, and congenital or early acquired brain lesion on MRI [23]. They had focal surgical resection or hemispherectomy despite abundant generalized or bilateral multifocal epileptiform discharges on preoperative EEG. Postsurgically, 72% of these patients achieved seizure freedom, 16% had marked improvement, 12% were not improved. Interestingly, the authors reported no significant differences in the rate of seizure-free outcome in association with age at seizure onset or surgery, presence of hemiparesis, or focal clinical features during seizures, type of lesion, or surgery type. In these cases, the consideration of epilepsy surgery is usually influenced by multiple factors including the presence of a unilateral or strongly asymmetric congenital or early-acquired lesion on neuroimaging, the severity of the refractory epilepsy, the low risk of incurring a new postoperative neurologic deficit, and in some patients the presence of localizing clinical features during seizures. The most striking age-related finding in this cohort was the age at occurrence of brain lesions. 90% of the lesions were congenital, perinatal, or acquired during infancy, predominantly malformations of cortical development, or cystic encephalomalacia. 

Lee et al. analyzed data of 27 children who had Lennox-Gastaut syndrome and underwent resective epilepsy surgery despite the presence of abundant generalized or generalized-contralateral maximal and multifocal epileptiform discharges on preoperative EEG [24]. 85% of these patients had identifiable lesions on brain MRI. At a mean of 33-month postoperative followup, 60% were seizure-free and another 15% had infrequent seizures. Interestingly, two out of four patients without brain abnormalities on MRI became seizure-free after resective surgery was performed on the basis of electrophysiologic studies and concordant results in other multimodal neuroimages. Most, 73%, of the reported patients showed an increase in developmental quotient after seizures declined.

A more recent study by Liu et al., of 18 patients with Lennox-Gastaut syndrome treated surgically showed similar results [25]. The authors reported good seizure outcome when majority of epileptiform discharges were ipsilateral to the brain lesion despite the presence of contralateral ictal discharges. Also they noted a better intellectual outcome with younger age at surgery or shorter interval between onset of seizures and resective operation [25]. 

Several other studies have indicated that, in carefully selected patients, early surgery during infancy or childhood may reduce serious social, psychological, and educational consequences of uncontrolled seizures and maximize functional recovery [26–30].

Considering the overall clinical picture in epileptic encephalopathy syndromes, one might raise the concern that the generalized or bilateral multifocal epileptiform discharges could be evidence that the lesion seen on neuroimaging is only “the tip of the iceberg” of a more diffuse epileptic process. This concern is supported by the presence of global developmental delay, the diffuse nature of some of the early brain insults such as perinatal intraventricular hemorrhage or infection with infarction, and in many cases the absence of focal clinical features during seizures. Features that may overcome this concern included the catastrophic nature of the epilepsy experienced by these devastated patients, the lack of good response to currently available nonsurgical treatments, the relatively low risk for new postoperative deficits in patients with preexisting hemiparesis, limited language development and/or poor functional level, and the characteristics of the lesion on preoperative MRI [23].

## 5. Conclusions

Early reports of successful surgery for selected children with infantile spasms and hypsarrhythmia were met with skepticism [31], but subsequent experience with similar clinical scenarios was supportive of this approach [7, 19, 21–25]. In catastrophic cases of epileptic encephalopathy, indications for surgery and assessment of its results require different rules from those that apply to adults and older children [32]. Epilepsy surgery in carefully selected patients may be effective in controlling seizures and improving neurological function despite the lack of localized epileptic pattern on EEG. It is expected that this new paradigm for pediatric epilepsy surgery will be refined in the future by additional clinical experience and further studies. 

## References

[1] Engel J (2001). A proposed diagnostic scheme for people with epileptic seizures and with epilepsy: report of the ILAE task force on classification and terminology. *Epilepsia*.

[2] Ohtahara S, Ishida T, Oka E, Yamatogi Y, Inoue H (1976). On the specific age-dependent epileptic syndrome: the early-infantile epileptic encephalopathy with suppression-burst. *No to Hattatsu*.

[3] Bernardina BD, Dulac O, Fejerman N (1983). Early myoclonic epileptic encephalopathy. *European Journal of Pediatrics*.

[4] Jeavons P, Livet MO, Roger J, Bureau M, Dravet C (1992). West syndrome: infantile spasms. *Epileptic Syndromes in Infancy, Childhood and Adolescence*.

[5] Vigevano F, Fusco L, Cusmai R (1993). The idiopathic form of West syndrome. *Epilepsia*.

[6] Hong AM, Turner Z, Hamdy RF, Kossoff EH (2010). Infantile spasms treated with the ketogenic diet: prospective single-center experience in 104 consecutive infants. *Epilepsia*.

[7] Kramer U, Sue WC, Mikati MA (1997). Focal features in West syndrome indicating candidacy for surgery. *Pediatric Neurology*.

[8] Dravet C, Bureau M, Guerrini R, Roger J, Bureau M, Dravet C (1992). Severe myoclonic epilepsy in infants. *Epileptic Syndromes in Infancy, Childhood and Adolescence*.

[9] Fukuma G, Oguni H, Shirasaka Y (2004). Mutations of neuronal voltage-gated Na^+^ channel *α*1 subunit Gene *SCN1A* in core severe myoclonic epilepsy in infancy (SMEI) and in borderline SMEI (SMEB). *Epilepsia*.

[10] Arzimanoglou A, French J, Blume WT (2009). Lennox-Gastaut syndrome: a consensus approach on diagnosis, assessment, management, and trial methodology. *The Lancet Neurology*.

[11] Heiskala H (1997). Community-based study of Lennox-Gastaut syndrome. *Epilepsia*.

[12] Chevrie JJ, Aicardi J (1972). Childhood epileptic encephalopathy with slow spike-wave. A statistical study of 80 cases. *Epilepsia*.

[13] Bureau M, Beaumanoir A, Bureau M, Deonna T, Mira L, Tassinari CA (1995). Continuous spikes and waves during slow sleep (CSWS): definition of the syndrome. *Continuous Spikes and Waves During Slow Sleep Or ESES*.

[14] Guerrini R, Genton P, Bureau M (1998). Multilobar polymicrogyria, intractable drop attack seizures, and sleep- related electrical status epilepticus. *Neurology*.

[15] Morrell F, Beaumanoir A, Bureau M, Deonna T, Mira L, Tassinari CA (1995). Electrophysiology of CSWS in Landau-Kleffner syndrome. *Continuous Spikes and Waves During Slow Sleep*.

[16] Landau WM, Kleffner FR (1957). Syndrome of acquired aphasia with convulsive disorder in children. *Neurology*.

[17] Kural Z, Ozer AF (2012). Epileptic encephalopathies in adults and childhood. *Epilepsy Research and Treatment*.

[18] Khan S, Al Baradie R (2012). Epileptic encephalopathies: an overview. *Epilepsy Research and Treatment*.

[19] Engel J, Cascino GD, Shields WD, Engel J, Pedley TA, Aicardi J (1998). Surgically remediable syndromes. *Epilepsy: A Comprehensive Textbook*.

[20] Morrell F (1989). Varieties of human secondary epileptogenesis. *Journal of Clinical Neurophysiology*.

[21] Wyllie E, Comair Y, Ruggieri P, Raja S, Prayson R (1996). Epilepsy surgery in the setting of periventricular leukomalacia and focal cortical dysplasia. *Neurology*.

[22] Chugani HT, Shields WD, Shewmon DA, Olson DM, Phelps ME, Peacock WJ (1990). Infantile spasms: i. PET identifies focal cortical dysgenesis in cryptogenic cases for surgical treatment. *Annals of Neurology*.

[23] Wyllie E, Lachhwani DK, Gupta A (2007). Successful surgery for epilepsy due to early brain lesions despite generalized EEG findings. *Neurology*.

[24] Lee YJ, Kang HC, Lee JS (2010). Resective pediatric epilepsy surgery in Lennox-Gastaut syndrome. *Pediatrics*.

[25] Liu S-Y, An N, Fang X (2012). Surgical treatment of patients with Lennox-Gastaut syndrome phenotype. *The Scientific World Journal*.

[26] Gleissner U, Sassen R, Schramm J, Elger CE, Helmstaedter C (2005). Greater functional recovery after temporal lobe epilepsy surgery in children. *Brain*.

[27] Berg AT (2004). UNderstanding the delay before epilepsy surgery: who develops intractable focal epilepsy and when?. *CNS Spectrums*.

[28] Freitag H, Tuxhorn I (2005). Cognitive function in preschool children after epilepsy surgery: rationale for early intervention. *Epilepsia*.

[29] Van Empelen R, Jennekens-Schinkel A, Van Rijen PC, Helders PJM, Van Nieuwenhuizen O (2005). Health-related quality of life and self-perceived competence of children assessed before and up to two years after epilepsy surgery. *Epilepsia*.

[30] Sabaz M, Lawson JA, Cairns DR (2006). The impact of epilepsy surgery on quality of life in children. *Neurology*.

[31] Hrachovy RA, Frost JD, Glaze DG, Kellaway P (1991). SUrgical treatment for infantile spasms?. *Annals of Neurology*.

[32] Aicardi J (1999). Evolution of epilepsy surgery in childhood: the neurologist's point of view. *Epileptic Disorders*.

